# Supplementary material for: Ester Linked Fatty Acid (ELFA) method should be used with caution for interpretating soil microbial communities and their relationships with environmental variables in forest soils
Source: PLoS One. 2021 May 10;16(5):e0251501. doi: 10.1371/journal.pone.0251501 (PMC8109784; doi:10.1371/journal.pone.0251501)
Supplement: S1 Table — (DOCX) [file pone.0251501.s001.docx]

**S1 Table** Climatic and soil characteristics at the six oriental oak (*Quercus variabilis*) forest sites in eastern China.

| **Selected**  **properties** | **Site** | | | | | |
| --- | --- | --- | --- | --- | --- | --- |
|  | **PG** | **HYS** | **BA** | **HZY** | **XY** | **YS** |
| Latitude (^o^N) | 40.25 | 39.48 | 37.09 | 34.02 | 32.12 | 29.09 |
| Longitude (^o^E) | 117.12 | 115.48 | 113.83 | 117.06 | 114.01 | 115.62 |
| Altitude (m) | 260 | 516 | 801 | 117 | 131 | 360 |
| MAP (mm) | 591 | 530 | 492 | 688 | 1045 | 1759 |
| MAT (^o^C) | 11.5 | 11.1 | 12.7 | 14.9 | 15.2 | 16.9 |
| Clay (%) | 23.3±3.1 | 21.0±1.3 | 12.7±0.4 | 35.0±0.4 | 14.8±0.4 | 28.3±0.0 |
| Silt (%) | 54.2±2.8 | 46.4±3.7 | 33.6±0.3 | 52.3±0.4 | 43.5±1.6 | 26.2±0.3 |
| Sand (%) | 22.5±0.3 | 32.6±3.0 | 53.7±0.6 | 12.7±0.4 | 41.7±1.9 | 45.6±0.3 |
| pH (in water) | 6.16±0.16 | 6.43±0.18 | 6.12±0.06 | 7.67±0.19 | 4.52±0.04 | 4.38±0.06 |
| SOC (%) | 2.40±0.14 | 3.24±0.25 | 3.22±0.04 | 2.10±0.02 | 3.34±0.05 | 3.83±0.13 |
| N (%) | 0.22±0.01 | 0.26±0.02 | 0.27±0.00 | 0.18±0.00 | 0.24±0.00 | 0.29±0.01 |
| C/N ratio | 10.64±0.17 | 11.93±0.18 | 11.38±0.12 | 11.59±0.21 | 13.28±0.14 | 13.17±0.21 |

MAP, mean annual precipitation; MAT, mean annual temperature. The climatic data is an average from 2010 to 2014 recorded by the local ecological stations. Values are mean ± standard error, n=3.
