# Supplementary material for: Ester Linked Fatty Acid (ELFA) method should be used with caution for interpretating soil microbial communities and their relationships with environmental variables in forest soils
Source: PLoS One. 2021 May 10;16(5):e0251501. doi: 10.1371/journal.pone.0251501 (PMC8109784; doi:10.1371/journal.pone.0251501)
Supplement: S2 Table — (DOCX) [file pone.0251501.s002.docx]

**S2 Table** Site-averaged absolute abundances of fatty acids (nmol g^-1^) for PLFA and ELFA methods

| **Fatty acid** | **Site PG** | | **Site HYS** | | **Site BA** | | **Site HZY** | | **Site XY** | | **Site YS** | |
| --- | --- | --- | --- | --- | --- | --- | --- | --- | --- | --- | --- | --- |
|  | **PLFA** | **ELFA** | **PLFA** | **ELFA** | **PLFA** | **ELFA** | **PLFA** | **ELFA** | **PLFA** | **ELFA** | **PLFA** | **ELFA** |
| i14:0 | 0.88 | 3.21 | 0.99 | 2.63 | 0.49 | 3.33 | 1.84 | 3.99 | 0.24 | 2.37 | 0.38 | 1.91 |
| C14:0 | 0.97 | 4.09 | 1.62 | 4.87 | 0.68 | 6.36 | 1.24 | 3.62 | 1.35 | 6.20 | 1.23 | 2.92 |
| i15:0 | 6.19 | 12.99 | 7.87 | 11.46 | 4.25 | 15.45 | 8.73 | 13.89 | 2.50 | 13.27 | 6.85 | 11.12 |
| a15:0 | 3.13 | 7.34 | 3.73 | 6.19 | 2.01 | 8.77 | 4.46 | 7.82 | 0.93 | 6.04 | 2.72 | 6.07 |
| C15:0 | 0.63 | 3.80 | 0.82 | 3.52 | 0.39 | 5.82 | 0.76 | 3.73 | 0.48 | 5.25 | 0.67 | 3.47 |
| i16:1 | 1.11 | 3.64 | 1.32 | 3.15 | 0.64 | 3.65 | 1.40 | 3.37 | 0.50 | 2.86 | 0.62 | 1.80 |
| i16:0 | 3.57 | 11.78 | 4.22 | 10.72 | 2.21 | 13.38 | 4.63 | 11.39 | 2.27 | 14.79 | 3.86 | 10.98 |
| a16:0 | 0.19 | 1.63 | 0.24 | 1.70 | 0.15 | 1.76 | 0.28 | 1.82 | 0.12 | 2.02 | 0.12 | 1.43 |
| 16:1ω9 | 0.88 | 2.53 | 1.30 | 2.59 | 0.58 | 3.06 | 1.57 | 3.13 | 0.25 | 2.04 | 0.55 | 1.51 |
| 16:1ω7 | 3.85 | 9.93 | 5.91 | 10.30 | 2.85 | 12.77 | 6.32 | 11.12 | 1.07 | 7.92 | 2.30 | 5.38 |
| 16:1ωx | 0.42 | 1.59 | 0.63 | 1.42 | 0.26 | 1.81 | 0.39 | 1.16 | 0.15 | 1.64 | 0.22 | 1.10 |
| 16:1ω5 | 2.13 | 10.61 | 3.64 | 11.98 | 1.53 | 12.41 | 4.22 | 12.50 | 0.58 | 10.13 | 1.43 | 8.01 |
| C16:0 | 11.43 | 34.49 | 15.08 | 38.45 | 8.03 | 52.08 | 13.55 | 31.30 | 9.85 | 47.31 | 12.14 | 26.80 |
| 17:1 | 2.58 | 13.11 | 4.33 | 13.79 | 1.43 | 20.43 | 8.68 | 14.05 | 0.42 | 20.48 | 1.49 | 12.30 |
| 10Me16:0 | 4.73 | 11.09 | 5.90 | 9.58 | 2.48 | 9.80 | 8.72 | 13.20 | 1.26 | 9.77 | 3.37 | 7.80 |
| br17:0 | 0.96 | 3.11 | 1.52 | 3.40 | 0.60 | 3.76 | 1.68 | 3.87 | 0.34 | 2.16 | 1.18 | 2.28 |
| i17:0 | 1.98 | 5.37 | 2.67 | 5.27 | 1.20 | 6.10 | 3.78 | 6.80 | 0.64 | 4.70 | 2.46 | 5.83 |
| a17:0 | 1.87 | 5.43 | 2.67 | 5.43 | 1.03 | 5.75 | 3.67 | 7.02 | 0.56 | 4.03 | 1.43 | 3.60 |
| 17:1ω7 | 0.81 | 4.70 | 1.13 | 4.51 | 0.51 | 6.29 | 1.09 | 4.49 | 0.29 | 3.92 | 0.37 | 2.48 |
| cy17:0 | 2.02 | 5.48 | 3.27 | 5.03 | 1.49 | 6.38 | 3.65 | 6.05 | 1.02 | 4.82 | 1.55 | 3.10 |
| C17:0 | 0.44 | 2.62 | 0.76 | 2.93 | 0.33 | 4.37 | 0.68 | 1.99 | 0.34 | 3.56 | 0.45 | 2.22 |
| br18:0 | 1.51 | 3.86 | 1.92 | 3.98 | 0.65 | 3.01 | 4.24 | 7.65 | 0.37 | 2.87 | 1.82 | 5.85 |
| br18:0 | 0.30 | 1.20 | 0.42 | 1.13 | 0.14 | 1.09 | 1.05 | 2.05 | 0.04 | 0.52 | 0.06 | 0.58 |
| 10Me17:0 | 0.81 | 3.86 | 0.97 | 3.53 | 0.47 | 4.40 | 0.91 | 3.35 | 0.47 | 4.01 | 0.94 | 3.67 |
| i18:0 | 0.32 | 1.41 | 0.33 | 1.54 | 0.19 | 1.43 | 0.42 | 1.63 | 0.17 | 1.48 | 0.43 | 1.20 |
| 18:3ω6 | 0.05 | 0.52 | 0.06 | 0.59 | 0.03 | 0.64 | 0.10 | 0.68 | 0.04 | 0.96 | 0.19 | 0.84 |
| 18:2ω9 | 0.26 | 1.39 | 0.38 | 1.68 | 0.22 | 1.92 | 0.42 | 1.72 | 0.12 | 1.81 | 0.12 | 1.02 |
| a18:0 | 0.17 | 1.91 | 0.21 | 3.62 | 0.09 | 1.37 | 0.33 | 2.65 | 0.10 | 3.18 | 0.22 | 2.91 |
| 18:2ω6 | 2.19 | 11.27 | 2.73 | 10.28 | 1.85 | 17.35 | 2.12 | 8.84 | 1.67 | 12.74 | 1.61 | 6.08 |
| 18:1ω9 | 8.13 | 24.40 | 10.23 | 23.70 | 4.92 | 34.38 | 9.75 | 21.80 | 5.57 | 22.82 | 7.01 | 15.97 |
| 18:1ω7 | 9.48 | 19.37 | 14.04 | 20.52 | 6.49 | 24.08 | 12.18 | 18.90 | 3.06 | 14.87 | 6.70 | 9.97 |
| 18:1ωx | 0.45 | 4.58 | 0.51 | 5.88 | 0.20 | 9.53 | 0.42 | 5.03 | 0.31 | 13.02 | 0.74 | 6.81 |
| 18:1ω5 | 0.88 | 2.55 | 1.74 | 2.86 | 0.48 | 2.63 | 2.03 | 3.46 | 0.12 | 1.41 | 0.41 | 1.09 |
| C18:0 | 3.42 | 10.90 | 4.57 | 11.42 | 2.09 | 15.72 | 4.56 | 10.30 | 2.72 | 12.92 | 3.94 | 9.74 |
| br19:1 | 1.35 | 4.23 | 2.12 | 4.28 | 1.27 | 5.53 | 1.90 | 4.05 | 0.42 | 4.34 | 1.18 | 3.18 |
| 10me18:0 | 2.94 | 9.65 | 3.39 | 9.00 | 1.32 | 11.62 | 5.85 | 8.96 | 0.55 | 12.24 | 1.67 | 8.29 |
| br19:0 | 0.53 | 1.88 | 0.67 | 1.84 | 0.26 | 1.87 | 1.01 | 2.23 | 0.10 | 1.63 | 0.45 | 2.40 |
| 19:1ω9 | 9.45 | 20.05 | 12.29 | 18.13 | 4.76 | 17.22 | 12.58 | 19.84 | 3.64 | 21.75 | 15.12 | 22.57 |
| C20:4 | 0.34 | 1.64 | 0.41 | 1.83 | 0.19 | 2.31 | 0.50 | 2.24 | 0.24 | 2.22 | 0.37 | 1.57 |
| C20:5 | 0.34 | 1.96 | 0.54 | 1.94 | 0.16 | 3.11 | 0.45 | 1.57 | 0.18 | 3.62 | 0.46 | 2.33 |
| C20:0 | 0.73 | 7.32 | 1.22 | 8.43 | 0.51 | 9.70 | 0.72 | 6.47 | 0.38 | 10.86 | 0.87 | 7.03 |
| C22:0 | 1.51 | 7.58 | 2.39 | 8.51 | 0.92 | 14.37 | 0.94 | 5.25 | 1.28 | 10.49 | 2.13 | 6.14 |
| C24:0 | 0.99 | 5.00 | 1.34 | 4.96 | 0.79 | 8.88 | 0.77 | 3.70 | 1.63 | 4.86 | 1.30 | 3.92 |
| Total | 96.94 | 305.07 | 132.11 | 308.58 | 61.16 | 395.69 | 144.58 | 308.68 | 48.35 | 339.86 | 93.12 | 245.30 |
