# Supplementary material for: Ester Linked Fatty Acid (ELFA) method should be used with caution for interpretating soil microbial communities and their relationships with environmental variables in forest soils
Source: PLoS One. 2021 May 10;16(5):e0251501. doi: 10.1371/journal.pone.0251501 (PMC8109784; doi:10.1371/journal.pone.0251501)
Supplement: S3 Table — (DOCX) [file pone.0251501.s003.docx]

**S3 Table** Site-averaged relative abundances of fatty acids (%) for PLFA and ELFA methods

| **Fatty acid** | **Site PG** | | **Site HYS** | | **Site BA** | | **Site HZY** | | **Site XY** | | **Site YS** | |
| --- | --- | --- | --- | --- | --- | --- | --- | --- | --- | --- | --- | --- |
|  | **PLFA** | **ELFA** | **PLFA** | **ELFA** | **PLFA** | **ELFA** | **PLFA** | **ELFA** | **PLFA** | **ELFA** | **PLFA** | **ELFA** |
| i14:0 | 0.91 | 1.05 | 0.75 | 0.85 | 0.79 | 0.84 | 1.27 | 1.29 | 0.50 | 0.70 | 0.40 | 0.78 |
| C14:0 | 1.01 | 1.34 | 1.23 | 1.58 | 1.12 | 1.60 | 0.86 | 1.17 | 2.80 | 1.82 | 1.32 | 1.19 |
| i15:0 | 6.36 | 4.27 | 5.94 | 3.72 | 6.95 | 3.90 | 6.03 | 4.50 | 5.17 | 3.90 | 7.35 | 4.53 |
| a15:0 | 3.21 | 2.41 | 2.82 | 2.01 | 3.28 | 2.21 | 3.08 | 2.53 | 1.92 | 1.77 | 2.93 | 2.48 |
| C15:0 | 0.65 | 1.24 | 0.63 | 1.14 | 0.65 | 1.47 | 0.53 | 1.21 | 0.99 | 1.54 | 0.72 | 1.41 |
| i16:1 | 1.14 | 1.19 | 1.00 | 1.02 | 1.05 | 0.92 | 0.97 | 1.09 | 1.02 | 0.85 | 0.66 | 0.73 |
| i16:0 | 3.68 | 3.86 | 3.20 | 3.47 | 3.62 | 3.38 | 3.21 | 3.69 | 4.74 | 4.37 | 4.13 | 4.47 |
| a16:0 | 0.19 | 0.53 | 0.18 | 0.55 | 0.25 | 0.45 | 0.20 | 0.59 | 0.24 | 0.60 | 0.13 | 0.58 |
| 16:1ω9 | 0.90 | 0.83 | 0.98 | 0.84 | 0.95 | 0.77 | 1.08 | 1.01 | 0.52 | 0.60 | 0.59 | 0.62 |
| 16:1ω7 | 3.95 | 3.25 | 4.46 | 3.34 | 4.66 | 3.22 | 4.36 | 3.60 | 2.23 | 2.34 | 2.47 | 2.20 |
| 16:1ωx | 0.43 | 0.52 | 0.48 | 0.46 | 0.43 | 0.45 | 0.27 | 0.38 | 0.32 | 0.48 | 0.24 | 0.45 |
| 16:1ω5 | 2.19 | 3.48 | 2.76 | 3.88 | 2.51 | 3.13 | 2.91 | 4.05 | 1.20 | 2.97 | 1.54 | 3.28 |
| C16:0 | 11.85 | 11.31 | 11.43 | 12.46 | 13.14 | 13.11 | 9.38 | 10.14 | 20.40 | 13.85 | 13.03 | 10.91 |
| 17:1 | 2.67 | 4.29 | 3.32 | 4.48 | 2.34 | 5.16 | 6.00 | 4.55 | 0.88 | 6.01 | 1.60 | 5.02 |
| 10Me16:0 | 4.88 | 3.65 | 4.45 | 3.10 | 4.05 | 2.50 | 6.03 | 4.28 | 2.61 | 2.91 | 3.61 | 3.17 |
| br17:0 | 0.99 | 1.02 | 1.15 | 1.10 | 0.98 | 0.95 | 1.16 | 1.26 | 0.71 | 0.64 | 1.26 | 0.93 |
| i17:0 | 2.03 | 1.76 | 2.02 | 1.70 | 1.96 | 1.54 | 2.62 | 2.20 | 1.32 | 1.39 | 2.63 | 2.37 |
| a17:0 | 1.92 | 1.78 | 2.02 | 1.76 | 1.68 | 1.45 | 2.54 | 2.27 | 1.15 | 1.18 | 1.54 | 1.47 |
| 17:1ω7 | 0.84 | 1.54 | 0.85 | 1.46 | 0.84 | 1.59 | 0.75 | 1.45 | 0.60 | 1.15 | 0.40 | 1.01 |
| cy17:0 | 2.08 | 1.80 | 2.47 | 1.63 | 2.43 | 1.61 | 2.52 | 1.96 | 2.10 | 1.41 | 1.66 | 1.26 |
| C17:0 | 0.46 | 0.86 | 0.57 | 0.95 | 0.54 | 1.10 | 0.47 | 0.64 | 0.70 | 1.05 | 0.49 | 0.90 |
| br18:0 | 1.56 | 1.27 | 1.45 | 1.29 | 1.06 | 0.77 | 2.94 | 2.48 | 0.76 | 0.85 | 1.95 | 2.38 |
| br18:0 | 0.31 | 0.39 | 0.32 | 0.37 | 0.23 | 0.28 | 0.72 | 0.66 | 0.08 | 0.15 | 0.06 | 0.24 |
| 10Me17:0 | 0.84 | 1.26 | 0.74 | 1.14 | 0.77 | 1.11 | 0.63 | 1.09 | 0.97 | 1.20 | 1.00 | 1.49 |
| i18:0 | 0.33 | 0.46 | 0.25 | 0.50 | 0.31 | 0.36 | 0.29 | 0.53 | 0.35 | 0.44 | 0.46 | 0.49 |
| 18:3ω6 | 0.05 | 0.17 | 0.05 | 0.19 | 0.04 | 0.16 | 0.07 | 0.22 | 0.08 | 0.28 | 0.21 | 0.34 |
| 18:2ω9 | 0.26 | 0.46 | 0.29 | 0.54 | 0.36 | 0.49 | 0.29 | 0.56 | 0.25 | 0.53 | 0.13 | 0.42 |
| a18:0 | 0.18 | 0.63 | 0.16 | 1.18 | 0.15 | 0.35 | 0.23 | 0.86 | 0.20 | 0.95 | 0.24 | 1.19 |
| 18:2ω6 | 2.25 | 3.69 | 2.08 | 3.34 | 3.02 | 4.40 | 1.47 | 2.86 | 3.44 | 3.73 | 1.74 | 2.48 |
| 18:1ω9 | 8.43 | 8.00 | 7.72 | 7.68 | 8.05 | 8.73 | 6.74 | 7.06 | 11.56 | 6.68 | 7.52 | 6.50 |
| 18:1ω7 | 9.74 | 6.35 | 10.61 | 6.64 | 10.61 | 6.06 | 8.41 | 6.12 | 6.29 | 4.38 | 7.21 | 4.07 |
| 18:1ωx | 0.48 | 1.51 | 0.39 | 1.91 | 0.33 | 2.40 | 0.29 | 1.63 | 0.64 | 3.81 | 0.79 | 2.77 |
| 18:1ω5 | 0.91 | 0.84 | 1.32 | 0.92 | 0.78 | 0.67 | 1.41 | 1.12 | 0.25 | 0.42 | 0.44 | 0.45 |
| C18:0 | 3.56 | 3.57 | 3.46 | 3.70 | 3.41 | 3.96 | 3.16 | 3.33 | 5.62 | 3.80 | 4.24 | 3.97 |
| br19:1 | 1.39 | 1.38 | 1.60 | 1.39 | 2.08 | 1.39 | 1.31 | 1.31 | 0.86 | 1.28 | 1.27 | 1.30 |
| 10me18:0 | 3.04 | 3.16 | 2.56 | 2.93 | 2.16 | 2.95 | 4.05 | 2.90 | 1.15 | 3.62 | 1.79 | 3.38 |
| br19:0 | 0.55 | 0.61 | 0.51 | 0.60 | 0.43 | 0.48 | 0.70 | 0.72 | 0.21 | 0.48 | 0.49 | 0.98 |
| 19:1ω9 | 9.74 | 6.57 | 9.29 | 5.87 | 7.79 | 4.37 | 8.71 | 6.43 | 7.48 | 6.37 | 16.24 | 9.20 |
| C20:4 | 0.35 | 0.54 | 0.31 | 0.59 | 0.31 | 0.58 | 0.35 | 0.73 | 0.49 | 0.65 | 0.39 | 0.64 |
| C20:5 | 0.36 | 0.64 | 0.41 | 0.63 | 0.26 | 0.79 | 0.31 | 0.51 | 0.38 | 1.07 | 0.49 | 0.95 |
| C20:0 | 0.75 | 2.40 | 0.93 | 2.74 | 0.83 | 2.45 | 0.50 | 2.10 | 0.78 | 3.21 | 0.94 | 2.87 |
| C22:0 | 1.55 | 2.49 | 1.80 | 2.75 | 1.50 | 3.65 | 0.65 | 1.70 | 2.65 | 3.12 | 2.28 | 2.51 |
| C24:0 | 1.03 | 1.63 | 1.02 | 1.61 | 1.29 | 2.25 | 0.53 | 1.20 | 3.39 | 1.45 | 1.40 | 1.60 |
